# Exposure to Atmospheric Particulate Matter Enhances Th17 Polarization through the Aryl Hydrocarbon Receptor

**DOI:** 10.1371/journal.pone.0082545

**Published:** 2013-12-11

**Authors:** Michael van Voorhis, Samantha Knopp, Walker Julliard, John H. Fechner, Xiaoji Zhang, James J. Schauer, Joshua D. Mezrich

**Affiliations:** 1 Department of Surgery, Division of Transplantation Surgery, University of Wisconsin School of Medicine and Public Health, Madison, Wisconsin, United States of America; 2 Department of Civil and Environmental Engineering, University of Wisconsin-Madison, Madison, Wisconsin, United States of America; McGill University, Canada

## Abstract

Lung diseases, including asthma, COPD, and other autoimmune lung pathologies are aggravated by exposure to particulate matter (PM) found in air pollution. IL-17 has been shown to exacerbate airway disease in animal models. As PM is known to contain aryl hydrocarbon receptor (AHR) ligands and the AHR has recently been shown to play a role in differentiation of Th17 T cells, the aim of this study was to determine whether exposure to PM could impact Th17 polarization in an AHR-dependent manner. This study used both cell culture techniques and *in vivo* exposure in mice to examine the response of T cells to PM. Initially experiments were conducted with urban dust particles from a standard reference material, and ultimately repeated with freshly collected samples of diesel exhaust and cigarette smoke. The readout for the assays was increased T cell differentiation as indicated by increased generation of IL-17A in culture, and increased populations of IL-17 producing cells by intracellular flow cytometry. The data illustrate that Th17 polarization was significantly enhanced by addition of urban dust in a dose dependent fashion in cultures of wild-type but not AHR^-/-^ mice. The data further suggest that polycyclic aromatic hydrocarbons played a primary role in this enhancement. There was both an increase of Th17 cell differentiation, and also an increase in the amount of IL-17 secreted by the cells. In summary, this paper identifies a novel mechanism whereby PM can directly act on the AHR in T cells, leading to enhanced Th17 differentiation. Further understanding of the molecular mechanisms responsible for pathologic Th17 differentiation and autoimmunity seen after exposure to pollution will allow direct targeting of proteins involved in AHR activation and function for treatment of PM exposures.

## Introduction

 Epidemiological studies have established a convincing connection between exposures to atmospheric particulate matter (PM) and increased morbidity and mortality due to airway disease. Exposure to air pollutants has been correlated with increases in the incidence and severity of asthma [1,2], chronic obstructive pulmonary disease (COPD) [3], respiratory infection [4] and even the rejection of lung allografts [5,6]. Multiple mechanisms to explain these effects of PM on pulmonary disease have been proposed and include PM acting as an adjuvant to allergens [7], the induction of the oxidative stress pathways [8], epigenetic gene regulation [9], and the induction of pro-inflammatory cytokines/chemokines by alveolar macrophages, dendritic cells or pulmonary epithelial cells [10].

 Interleukin-17A (IL-17A) is a cytokine generated by T cells as part of the host defense to bacterial and fungal infections. IL-17A has also been implicated as an important component of airway diseases including asthma [11] and COPD [12] as well as the rejection of lung transplants [13,14]. In various mouse models, exposure to PM *in vivo* has been shown to upregulate IL-17 expression in the lung [15,16] or the gut [17]. 

 Recently, several labs have described a role for the aryl hydrocarbon receptor (AHR) in the regulation of Th17 differentiation [18–20]. In these studies, the AHR ligand 6-formylindolo[3,2-b]carbazole (FICZ), a tryptophan photoproduct, enhanced the Th17 response in naïve T cells and promoted autoimmunity in a murine model. IL-22, a cytokine associated with but not exclusive to Th17 cells, was also shown to be highly regulated through the AHR.

 The AHR has been considered the primary receptor for polycyclic aromatic hydrocarbons (PAHs) [21], which are present in the environment and inhaled into the lung secondary to exposures to PM from cigarette smoke, auto emissions, industrial exhaust, burning wood and charcoal, and urban dust. Given that the lung is in direct contact with the atmosphere, components of PM, in particular PAHs, may act on T cells to alter immune responses towards an inflammatory response. To test this hypothesis, a standardized PM sample, SRM1649b, was added to Th17 cultures. The results show that PM can enhance Th17 responses in an AHR-dependent manner. Further evidence demonstrates that PAHs contained in the PM are likely sources of Th17-enhancing activity. These findings have ramifications in understanding the mechanisms of airway pathology caused by environmental exposures, and could ultimately delineate the AHR as a target for intervention to prevent or treat environmentally-induced diseases. 

## Materials and Methods

### Mice

C57BL/6 and Balb/c wild-type mice were obtained from Jackson Laboratories. Christopher Bradfield provided AHR null (AHR^-/-^) [22] and DREC [23] mice, both on a C57BL/6 background. All mice were maintained under specific, pathogen-free conditions. All animal experiments were performed in accordance with protocols approved by the School of Medicine and Public Health (SMPH) Institutional Animal Care and Use Committee at the University of Wisconsin-Madison. 

### Intranasal administration of SRM1649b and RNA isolation from murine lungs

To model exposure to ambient urban particulate matter, Standard Reference Material (SRM) 1649b Urban Dust was obtained from the National Institute of Standards and Technology (NIST; Gaithersburg, MD). The certificate of analysis for SRM1649b used in this study is available online. Dispersed suspensions of SRM1649b were created by sonication in sterile PBS for 15 minutes in a cooking water bath. Endotoxin contamination was determined using the LAL Chromogenic Endotoxin Quantization kit from Pierce (Rockford, IL), and endotoxin contamination of a stock suspension of 20mg/ml SRM1649b was below the level of detection (0.1 EU/ml). For intranasal administration of SRM1649b, mice were anesthetized using isoflurane, and 20μL of 20mg/mL SRM1649b or 40μL PBS was introduced via nasal inhalation on days 0, 3 and 6. Mice were sacrificed on day 7 and their lungs were harvested for RNA isolation (Qiagen) and RT-PCR. 

### Isolation of Naïve T Cells and T Cell Differentiation

 Naïve CD4^+^ T cells were purified from C57BL/6, AHR wild-type, Het, KO or other transgenic male mouse spleens by using the CD4^+^ Isolation Kit and CD62L MicroBeads (Miltenyi) in conjunction with an AutoMacs separator. Purified naïve T cells were stimulated with the T cell CD3/CD28 cell Activation/Expander Kit (Miltenyi) for 3 days. As indicated, mouse IL-6 (20 ng/mL; R&D Systems), mouse TGF-β (5 ng/mL; R&D Systems), FICZ (200 nM; Enzo Life Sciences), or SRM1649b (NIST) were added to cultures. Media used for cultures was RPMI 1640 supplemented with Hepes, non-essential amino acids, sodium pyruvate (Cell Gro), glutamine, penicillin/streptomycin, and 10% FCS (Hyclone). In mixed lymphocyte reactions, DCs were isolated from allo- (balb/c) or syngeneic- (C57BL/6) generic murine spleens with CD11c immunomagnetic beads (Miltenyi) and co-cultured with isolated naïve T cells in indicated conditions. 

### Quantitative Real Time PCR (qRT-PCR)

 Total RNA was extracted using the RNeasy Mini Kit and RNase-Free DNase Set (Qiagen). 500 ng of total RNA in each group was used for cDNA sythesis (iScript cDNA Synthesis Kit, BioRad or High-capacity cDNA Reverse Transcription Kits, Life Technologies). The qRT-PCR was performed on the Applied Biosystems 7900HT Fast Real-Time PCR System using Taqman Gene Expression Assays and TagMan Universal PCR Master Mix (Life Technologies). Taqman Gene Expression Assays used include: actin b, Mm01205647_g1; IL-17A, Mm00439619_m1; IL-22, Mm00444241_m1; Cyp1A1, Mm00487218_m1; Cyp 1B1, Mm00487229_m1; IL-23R, Mm00519943_m1 and AHR, Mm00478932_m1. Data was analyzed using the ΔΔCt method with actin serving as the endogenous reference.

### IL-17, IL-22, IFN-γ ELISAs and Intracellular Cytokine Staining

 Supernatant was collected and analyzed for mouse IL-17, IL-22, and IFN-γ by ELISA according to manufacturer’s instructions (R&D Systems). For intracellular cytokine staining, cultured cells were stimulated with Cell Stimulation Cocktail (eBioscience) for 5 hours. Brefeldin A (BD PharMingen) was added for the final 4 hours. Cells were then fixed and permeabilized with Intracellular Fixation & Permeabilization Buffer (eBioscience). Cells were stained with PE- conjugated AHR, AlexaFluor 488-conjugated anti–IFN-γ, and eFluor660- conjugated anti–IL-17 as well as anti-CD4 and anti-CD3 fluorochrome conjugated antibodies. In some experiments, cells were stained prior to fixation with Fixable Viability Dye eFluor 450. All antibodies and live dead stains were from eBioscience. Stained cells were analyzed on either a FACSCalibur, LSRII or Accuri C6 (all from BD). Data was analyzed using FlowJo software (TreeStar).

### Fractionation of SRM1649b and Gas Chromatography-Mass Spectrometry (GC-MS)

 119 mg of SRM1649b was extracted in dichloromethane (DCM) for 24 hours in a Soxhlet extractor. The sample was placed in a rotovap and reduced in volume to 5 mL and an aliquot was set aside for GC-MS analysis. The remaining sample was transferred to a test tube and blown down under nitrogen and quantitatively solvent exchanged with DMSO. The aliquot set aside for GCMS analysis was analyzed using the solvent extraction method as previously described [24]. PAHs, hopanes, and steranes were quantified using authentic standards and deuterated internal standards using an Agilent 5973 GCMS and an HP-5MS GC column. 

### Sample collection methods

 Cigarette and Wood Smoke: Cigarette and wood smoke particulate matter (PM) samplers were generated in the laboratory using a Filimatic vial filter (#17723) automated smoking machine and a BioLite Campstove. The combustion devices were separately placed in a sealed residence time chamber approximately one-meter square and allowed to equilibrate with continuous combustion of the fuel material (commercially available regular filter cigarettes and pelletized wood fuel), the chamber was completely mixed through the use of an internal mixing fan placed inside the chamber. Primary dilution air, scrubbed with activated carbon, desiccant dried, and HEPA filtered, was cycled through the chamber at a rate of 50 LPM. PM mass was collected from the exhaust of the chamber on Teflon and quartz filters after passing through a PM2.5 size selective cyclone at a sample rate of 24 LPM (8 LPM per filter channel). Excess sample air was exhausted out of the chamber to a fume hood. The chamber was evacuated both before and after each combustion event with scrubbed dilution air so that particle number in the chamber was below 10 particles per cm^3^ as measured by a condensation particle counter (CPC, TSI 3010). 

 Diesel Exhaust: A detached diesel refrigeration unit (ThermoKing with Yanmar Diesel Engine), as typically used for temperature control in tractor-trailer box vans, was operated with commercial available Ultra-Low sulfur diesel (ULSD) at steady state, high RPM and design load for maximum cooling. A dilution sampler was used to dilute the engine exhaust with filtered and scrubbed ambient air to mimic real atmospheric dilution of engine exhaust. The sampler was the same as previously described [25] and applied to emissions testing as previously described [26]. Samples were collected on 47 mm quartz filters after a PM2.5 cyclone with a sample flow rate of 24 LPM (12 LPM per filter channel). 

### Statisitics

 Statistical analysis was performed using GraphPad Prism software. With the exception of ELISA data in which data in control samples was below the limits of assay detection, data was analyzed using two-way ANOVA with Bonferonni post-tests. For data sets which included data below the level of detection, Fisher’s exact test was utilized.

## Results

### Exposure to SRM1649b augments IL-17 expression *in vivo* and *in vitro*

**Figure 1:**
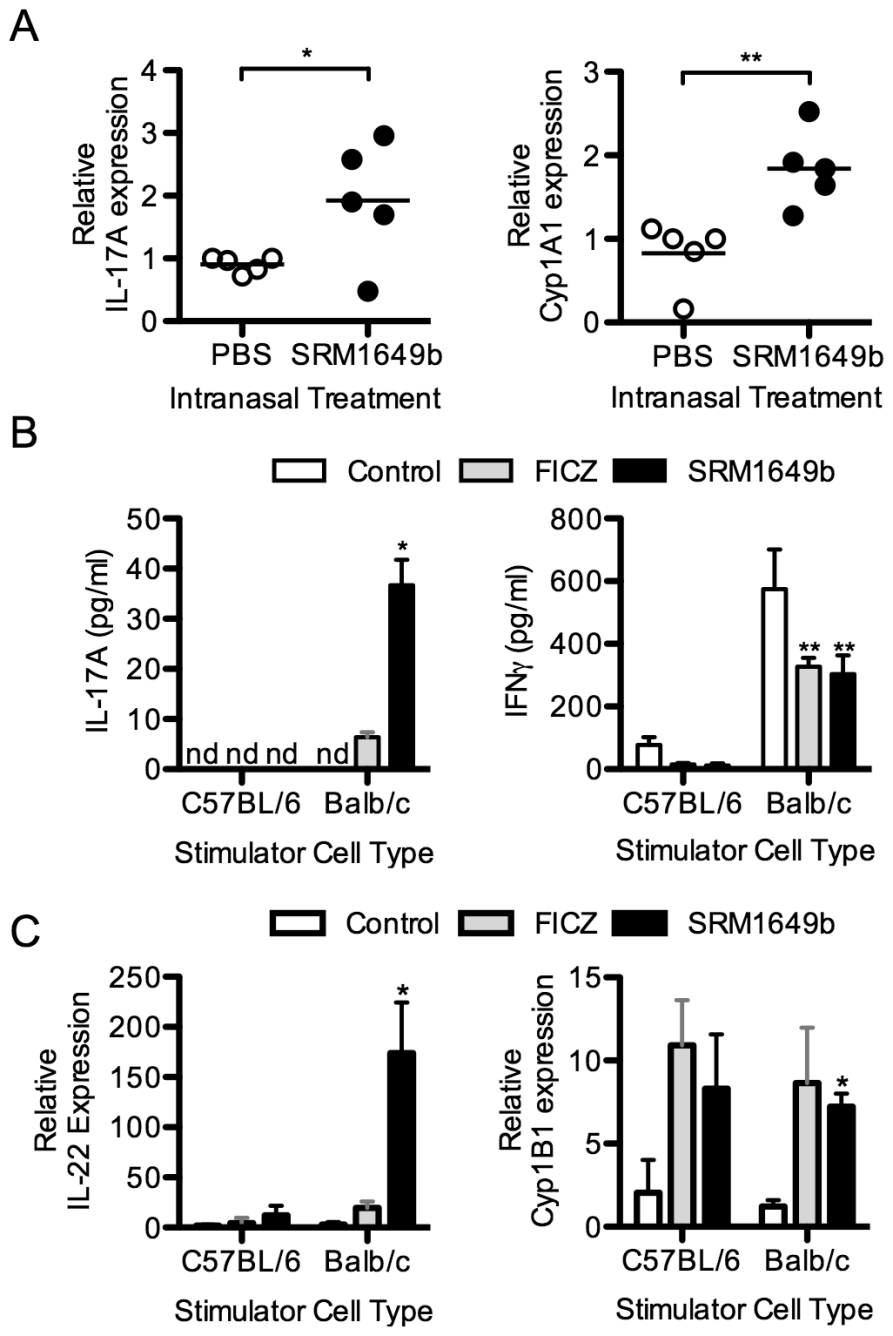
Exposure to SRM1649b augments IL-17 expression *in* *vivo* and *in* *vitro*. A: Male C57BL/6 mice (B6) were exposed every 3 days to SRM1649b or control PBS (400 μg/20μl PBS) by intranasal administration. 24 hours after the final treatment, mice were euthanized and total RNA was isolated from the right lung. qRT-PCR was performed to measure changes in the steady state expression of IL-17A and Cyp1a1 mRNA. B-C: Splenocytes from male B6 mice were stimulated *in* *vitro* with dendritic cells isolated from B6 or Balb/c splenocytes in the presence or absence (Control) of either FICZ (200 nM) or SRM1649b (40 μg/ml). After 4 days, supernatants were harvested and the concentration of IL-17A and IFN-γ was measured by cytometric bead array. Total RNA was also isolated from the cultured cells and the relative changes in IL-22 and Cyp1b1 mRNA expression was measured by qRT-PCR. Data from 3 separate experiments were pooled and statistical comparison was made between controls and experimental cultures. *, p < 0.05; **, p < 0.01;.

 To investigate whether exposure to SRM1649b could influence IL-17A expression levels *in vivo*, preliminary experiments were performed on C57BL/6 (B6) mice. Mice were treated doses of SRM1649b stock solution (20 μl/day) or PBS intranasally every 3 days for a total of 3 doses. Twenty-four hours after the last dose, mice were sacrificed and the right lung was harvested for RNA isolation. SRM1649b-treated mice exhibited 2-fold higher IL-17A mRNA levels (Figure 1A). Evidence of AHR signaling was demonstrated by a similar increase in the cytochrome P450 enzyme gene cyp1a1 mRNA. 

 Because of an interest in the role of immune cells in response to PM, and recent data that PM plays a role in chronic lung rejection driven by IL-17 [5,6], the effects of SRM1649b were tested in murine mixed leukocyte cultures (MLC). These assays allow interrogation of the effects of SRM1649b on dendritic cells (DC) and T cells in an alloimmune setting, as might be seen after a lung transplant. In co-cultures of B6 splenocytes and Balb/c dendritic cells (DC) (alloimmune response), IL-17A levels increased in the supernatant by ELISA in MLC containing FICZ (it was undetectable in the control wells) while the addition of 40 µg/ml SRM1649b increased IL-17A 8-fold higher still (Figure 1B). There was no IL-17 detected in syngeneic wells (T cells and DCs from B6 mice). Interferon-gamma (IFN-γ) levels were decreased by approximately 50% by both FICZ and SRM1649b. The addition of FICZ or SRM1649b up regulated IL-22 mRNA expression in both syngeneic and allogeneic cultures, though the increase was substantially higher in the allogeneic cultures containing SRM1649b (Figure 1C). Similarly, cyp1b1 expression increased in both allogeneic and syngeneic cultures when either FICZ or SRM1649b were added to the cultures suggesting AHR activation had occurred in these cultures. 

### Th17 differentiation *in vitro* is enhanced with addition of SRM1649b to culture conditions

**Figure 2:**
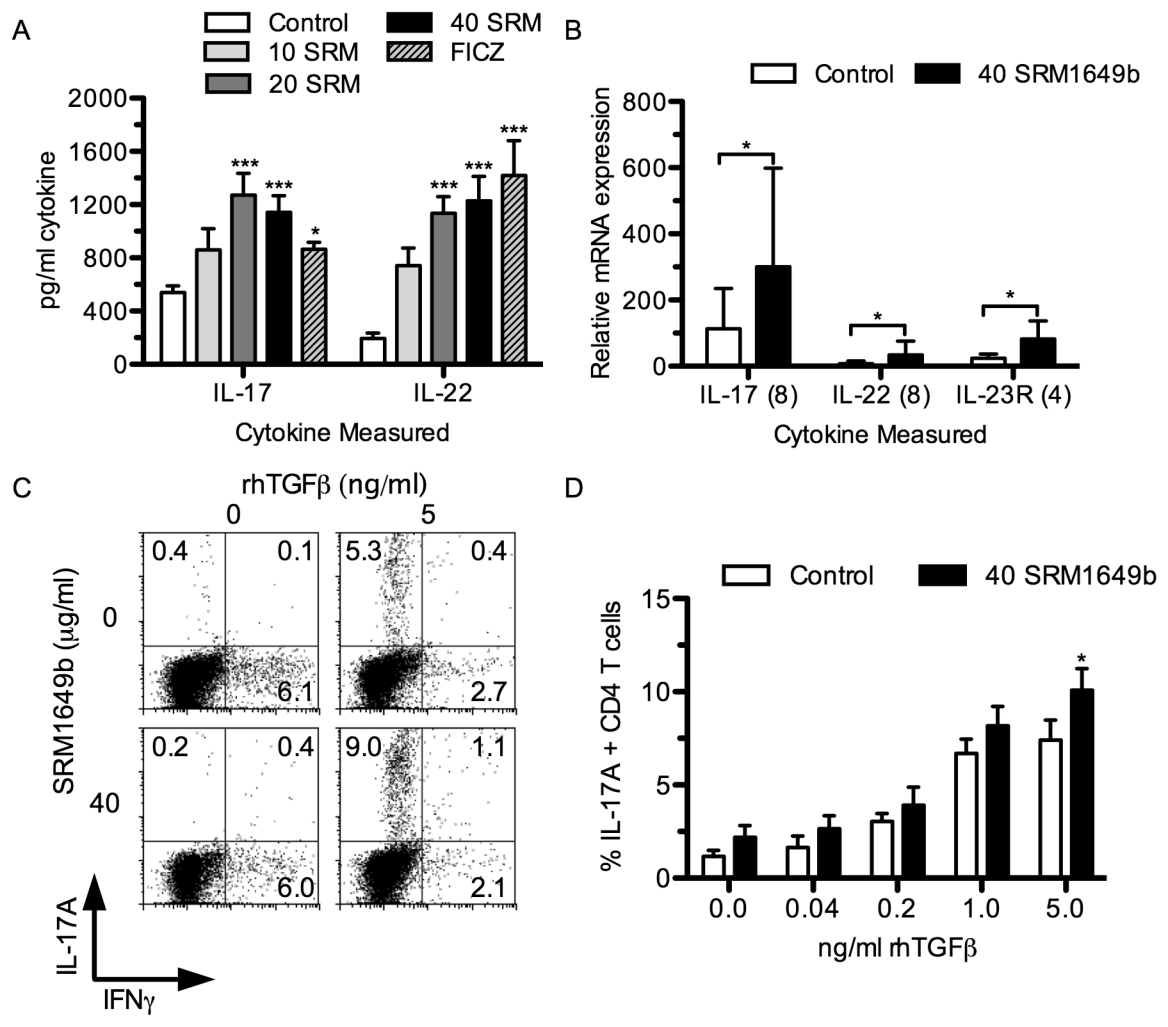
Th17 differentiation *in* *vitro* is enhanced with addition of SRM1649b to culture conditions. A-B: Naïve CD4^+^ T cells were isolated from the spleens of male C57BL/6 mice (B6) and cultured in Th17 conditions (anti-CD3/CD28 antibody + mIL-6 (20 ng/ml) + huTGF-β (5 ng/ml)) for 4 days in the absence or presence of SRM1649b (10, 20 or 40 μg/ml) or FICZ (200 nM). Triplicate culture supernatant was harvested and ELISA measured the concentration of IL-17A and IL-22 (A). Data presented is representative of 3 experiments. In separate experiments, total RNA was harvested from cells and the relative levels of IL-17A, IL-22 and IL-23R mRNA were determined by qRT-PCR (B). Data is relative to cells treated with anti-CD3/CD28 antibody stimulation but without mIL-6 and huTGF-β. Fold increase results from cultures with (w/) SRM1649b were compared for significance with cultures without (w/o) SRM1649b using the paired student’s t-test. The number in parenthesis indicates the number of individual assays used for that target gene. C-D: Naïve B6 CD4^+^ T cells were cultured in Th17 conditions (anti-CD3/CD28 antibody + mIL-6 (20 ng/ml) and varying concentrations of huTGF-β (0 - 5 ng/ml)) for 4 days in the absence or presence of SRM1649b (40 μg/ml). Cells were harvested from and subjected to intracellular cytokine analysis to determine the percent of IL-17A and IFN-γ expressing CD4^+^ T cells present after culture. Data from 3 separate experiments were pooled and statistical comparison was made between the cultures with versus cultures without SRM1649b at each concentration of huTGF-β. *, p < 0.05; **, p < 0.01; ***, p < 0.001.

 As AHR ligands have been shown to enhance Th17 differentiation *in vitro* [18], SRM1649b was added to naïve B6 CD4^+^ T cells cultured under Th17 polarizing conditions. After 4 days supernatant was harvested for analysis by ELISA. As can be seen in Figure 2A, doses of 20 and 40 µg/ml of SRM1649b consistently and significantly increased IL-17A as well as IL-22 levels in the supernatant. Using qRT-PCR, similar relative increases were found at the mRNA level for IL-17A and IL-22 (Figure 2B). As IL-23 is an important cytokine for the maintenance of Th17 activity [27], IL-23R mRNA levels were also analyzed and found to have increased by the addition of SRM1649b.

 While these data suggest that SRM1649b can increase the amount of IL-17 in culture, it does not address whether the effects were due to an increase in Th17 cell differentiation or increased expression of IL-17A cytokine. For this, intracellular cytokine analysis was used. As shown in Figure 2C and D, there was an increase in the percentage of IL-17-producing CD4^+^ T cells when SRM1649b was added to the cultures at all doses of TGF-β, which became significant at 5 ng/ml of TGF-β. The magnitude of increased Th17 differentiation was less than the increase in IL-17 mRNA and protein levels, suggesting that SRM1649b may both increase Th17 differentiation and enhance protein expression from the existing Th17 cells.

### AHR is required for upregulation of Th17 cells in response to SRM1649b

**Figure 3:**
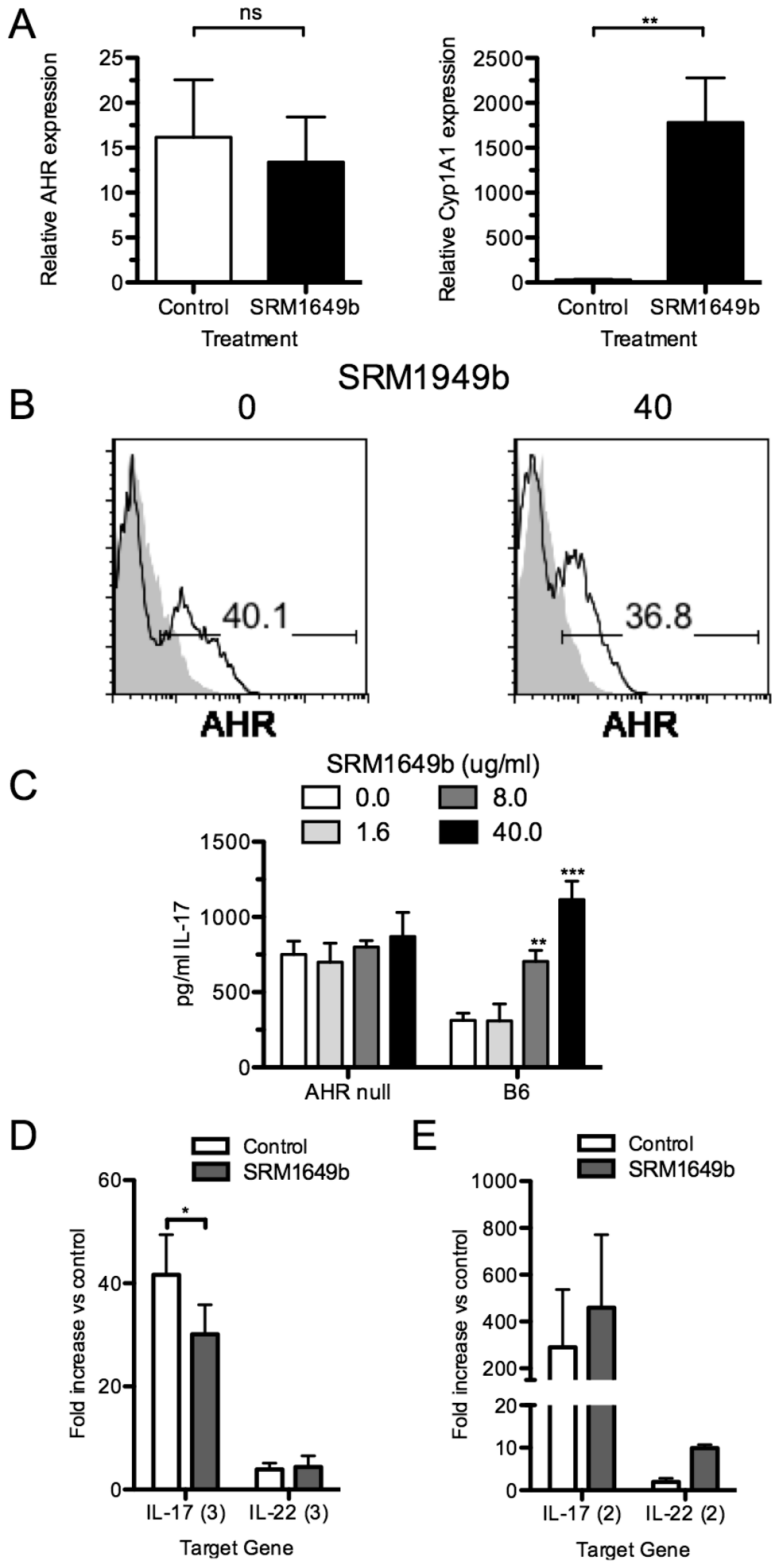
AHR is required for upregulation of Th17 cells in response to SRM1649b. A-B: Naïve B6 CD4^+^ T cells were cultured in Th17 conditions (anti-CD3/CD28 antibody + mIL-6 (20 ng/ml) and varying concentrations of huTGF-β (5 ng/ml) for 4 days in the absence or presence of SRM1649b (40 μg/ml) after which total RNA was harvested. Relative levels of AHR and cyp 1a1 mRNA were determined by qRT-PCR. Data are relative to cells treated with anti-CD3/CD28 antibody stimulation but without mIL-6 and huTGF-β. Fold increase results from cultures with (w/) SRM1649b were compared for significance with cultures without (w/o) SRM1649b using the paired student’s t-test. B: Cells were harvested after 4 days and stained with anti-AHR antibody during intracellular cytokine analysis to determine the percent of AHR-expressing CD4^+^ T cells cultured with (solid line) or without (shaded) huTGF-β. C: Naïve CD4^+^ T cells were isolated from the spleens of male B6 or AHR^-/-^ mice and cultured in Th17 conditions (anti-CD3/CD28 antibody + mIL-6 (20 ng/ml) +/- huTGF-β (5 ng/ml) for 4 days in the varying concentrations SRM1649b. Supernatants were analyzed by ELISA. D - E: Naïve CD4^+^ T cells were isolated from the spleens of AHR^-/-^ (D) or DREC^-/-^ (E) mice and cultured in Th17 conditions (anti-CD3/CD28 antibody + mIL-6 (20 ng/ml) +/- huTGF-β (5 ng/ml) for 4 days +/- SRM1649b. Total RNA was harvested and, the relative levels of IL-17A, and IL-22 mRNA were determined by qRT-PCR. Data is relative to cell treated with anti-CD3/CD28 antibody stimulation but without added mIL-6 and huTGF-β. Fold increase results from cultures with (w/) SRM1649b were compared for significance with cultures without (w/o) SRM1649b using the paired student’s t-test. The number in parenthesis indicates the number of individual assays used for that target gene. *, p < 0.05; **, p < 0.01; ***, p < 0.001.

 To explore whether the effects of SRM1649b on IL-17 expression *in vitro* are mediated via AHR activation versus an effect on expression of AHR, the ability of SRM1649b to alter AHR expression was examined at both the mRNA and protein level. While AHR expression did increase over baseline when cells were placed in Th17 polarizing condition measured by qRT-PCR, the addition of SRM1649b did not significantly change AHR mRNA expression (Figure 3A). Similarly, when examined by flow cytometry, the increase in AHR expression in CD4^+^ T cells seen with the addition of TGF-β (solid line, compared to shaded line without TGF-β) was not significantly altered by the addition of SRM1649b (Figure 3B). The percent of AHR^+^ CD4^+^ T cells was noted to be greater than the percent of IL-17A^+^ CD4^+^ T cells. Further investigation revealed that there is an increase in AHR staining both in IL-17^+^ cells and IFN-γ^+^ cells (Figure S1). In addition, a fraction of IL-17^-^/IFN-γ^-^ cells demonstrated increased AHR expression also. 

 While the data does not demonstrate that the Th17 polarization enhancement activity of SRM1649b is due to upregulation of the AHR, qRT-PCR for cyp1a1 mRNA expression strongly suggests AHR activation is occurring in cultures containing this sample PM (Figure 3A). To determine the role of the AHR in increased Th17 differentiation, naïve CD4^+^ T cells from AHR^-/-^ mice were cultured in Th17 polarizing conditions with increasing concentrations of SRM1649b (Figure 3C). At baseline, the amount of IL-17 in the culture supernatant was higher than seen with wild-type cells. Addition of SRM1649b did not increase IL-17 concentration in the supernatant in the null cultures, in contrast to a dose-dependent increase in wild-type cells with addition of SRM1649b (Figure 3C). IL-22 was not detected in AHR^-/-^ cultures (data not shown). The relative expression of IL-17A mRNA was decreased 25% when 40µg/ml SRM1649b was added to the AHR^-/-^ Th17 cultures (Figure 3D). 

 Recently, the activity of some presumed AHR ligands *in vitro* has been questioned by the finding that some “ligands” do not actually activate the AHR directly, but instead inhibit cytochrome P450 activity, in particular cyp1a1 activity. This can prevent the metabolism of AHR ligands present in culture media and give the appearance of AHR activation [28]. Furthermore, tryptophan breakdown products (including FICZ) can be found culture media and these products can enhance Th17 polarization in an AHR-dependent manner [29]. FICZ has been shown to be metabolized primarily by cyp1a1 activity [28,30]. Therefore, to investigate whether the Th17 differentiation activity found in SRM1649b was not a direct effect of components of SRM1649b on the AHR, but instead was due to cyp1a1 antagonism, naïve CD4^+^ T cells from DREC-null (DREC^-/-^) mice were utilized. DREC^-/-^ mice do not upregulate cyp1a1 and cyp1a2 in response to AHR ligands, as a cluster of dioxin-response elements (DREs) upstream of cyp1a1 and cyp1a2 has been deleted by homologous recombination. Cyp1a1 and cyp1a2 are consequently minimally expressed in these mice [23]. Two separate experiments were performed using naïve CD4^+^ T cells isolated from DREC^-/-^ mice cultured under Th17 conditions for 4 days in the absence or presence of SRM1649b (Figure 3E). CD4^+^ T cells from DREC^-/-^ mice showed a greater baseline increase in IL-17A mRNA compared to T cells from B6 mice (data not shown). This may be due to increased half-life of P450-labile AHR ligands within the media. Despite this increased baseline, DREC CD4^+^ T cells exhibited increased IL-17A mRNA expression when exposed to SRM1649b under Th17 conditions, though less than what was seen in B6 mice. An average 4-fold increase in IL-22 expression was also seen. As expected, cyp1a1 mRNA remained low in DREC^-/-^ T cells under all conditions (data not shown). These findings suggest that the Th17-enhancing activity found in SRM1649b was not due to inhibition of cyp1a1 or cyp1a2 activity. This further supports that these enzymes were not needed to metabolize SRM1649b to generate ligands involved in the enhancement of Th17 polarization. 

### Investigation of the specific components of SRM1649b that contain Th17 enhancing activity

**Figure 4:**
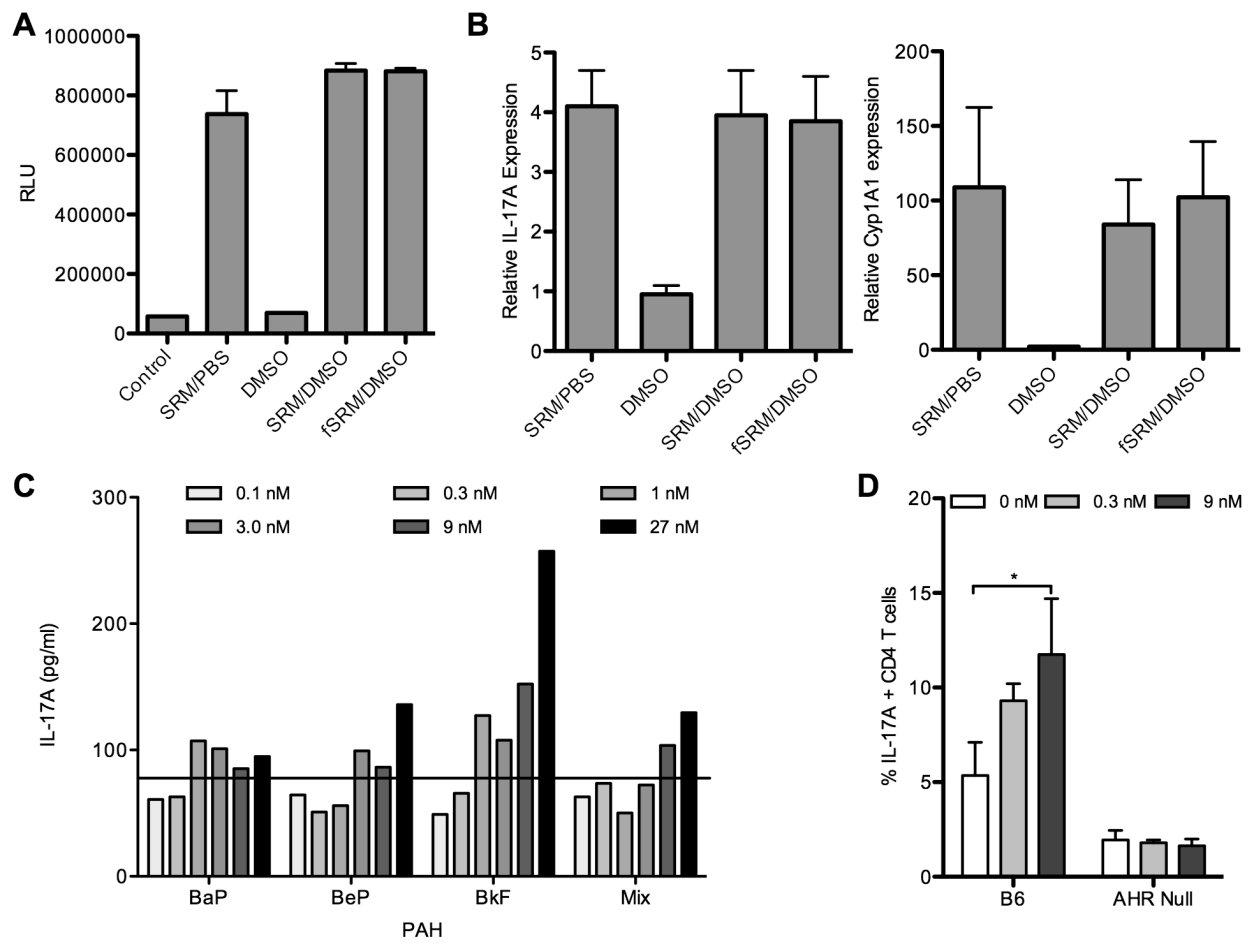
Investigation of the specific components of SRM1649b that contain Th17 enhancing activity. A: Mouse Hepa1 cells that were transfected with luciferase reporter gene fused to the dioxin-responsive elements (DRE) were seeded at 0.6x10^6^ cells per well. Cells were then exposed to media (Control), media + SRM1649b suspended in PBS (SRM/PBS), media + DMSO (DMSO), media + SRM1649b suspended in DMSO (SRM/DMSO) or the organic fraction of SRM1649b suspended in DMSO (fSRM/DMSO) for 4 hours. Luciferase activity was measured on a luminometer. B: Naïve B6 CD4^+^ T cells in Th17 conditions in the presence of fractioned SRM1649b and controls. After 4 days, total RNA was harvested. Using qRT-PCR, the relative levels of IL-17A and Cyp1a1 mRNA were determined. Data presented are the mean and standard deviation of 2 separate experiments and is relative to cells treated with anti-CD3/CD28 antibody stimulation but without mIL-6 and huTGF-β. C: Naïve B6 CD4^+^ T cells in Th17 conditions in the presence varying concentrations of the PAHs benzo[a]pyrene (BaP), benzo[e]pyrene (BeP) and benzo[k]fluoranthene (BkF). Culture supernatant was harvested and ELISA measured the concentration of IL-17A. The line indicates the level of IL-17A expression in cultures exposed to DMSO vehicle only. D: Naïve B6 or AHR^-/-^ CD4^+^ T cells in Th17 conditions in the presence of BkF. Cells were harvested and subjected to intracellular cytokine analysis to determine the percent of IL-17A-expressing CD4^+^ T cells present after culture. Data from 3 separate experiments was pooled and statistical comparison was made between the cultures with versus cultures without BkF.

 To establish whether the SRM1649b response was due to PAHs or related lipophylic compounds, fractionation of SRM1649b into its chemical constituencies was performed. As a first step, the organic extractable compartment was isolated using dichloromethane, drying down the extract and solubilizing the fractionated SRM1649b in DMSO. A luciferase-based assay system was utilized to demonstrate that the fractionated SRM1649b (fSRM) contained AHR activity (Figure 4A). When fSRM was utilized in Th17 assays the same level of AHR activation as the parental SRM1649b (as measured by qRT-PCR for cyp1a1), and a similar enhancement of IL-17A mRNA over Th17 conditions was seen (Figure 4B). Analysis of fSRM on gas chromatography-mass spectrometry (GC-MS) identified 21 PAHs, with concentrations ranging from 40-968 pg/µg in the mixture, which was similar to the PAHs identified by NIST in their analysis of SRM1649b (Table 1). Additionally 5 steranes and 11 hopanes were identified.

**Table 1:** Analysis of fractionated SRM1649b.

**Compound family**	**Compound**	**pg/μg**
**Polycyclic Aromatic Hydrocarbons**	1-Methylchrysene	**0**
	Cyclopenta(cd)pyrene	**0**
	Acephenanthrylene	**39.64**
	Retene	**56.02**
	Perylene	**80.89**
	Benzo(j)fluoranthene	**82.80**
	Anthracene	**118.38**
	Picene	**123.44**
	Dibenzo(ae)pyrene	**128.66**
	Benzo(GHI)fluoranthene	**151.13**
	Dibenz(ah)anthracene	**185.37**
	Benz(a)anthracene	**324.12**
	Benzo(a)pyrene	**416.92**
	Coronene	**548.29**
	Indeno(1,2,3-cd)pyrene	**581.11**
	Benzo(k)fluoranthene	**591.88**
	Chrysene	**637.16**
	Phenanthrene	**639.49**
	Benzo(e)pyrene	**651.53**
	Benzo(GHI)perylene	**676.68**
	Pyrene	**786.99**
	Benzo(b)fluoranthene	**962.49**
	Fluoranthene	**968.37**
**Steranes**	ABB-20R-C27-Cholestane	**157.00**
	ABB-20R-C28-Ergostane	**167.57**
	ABB-20S-C28-Ergostane	**179.42**
	AAA-20S-C27-Cholestane	**232.70**
	AAA-20R-C27-Cholestane	**255.89**
	17A(H)-22,29,30-Trisnorhopane	**308.28**
	ABB-20S-C29-Sitostane	**333.30**
	22R-Trishomohopane	**333.33**
	ABB-20R-C29-Sitostane	**383.79**
**Hopanes**	22S-Trishomohopane	**531.94**
	22R-Bishomohopane	**682.11**
	22S-Bishomohopane	**835.24**
	22R-Homohopane	**1099.69**
	17A(H)-21B(H)-30-Norhopane	**1365.41**
	22S-Homohopane	**1396.14**
	17A(H)-21B(H)-Hopane	**2103.26**

 PAHs are obvious candidates as the responsible components of SRM1649b that could enhance Th17 polarization, as they are known AHR ligands. The total concentration of PAHs in the 40µg/ml SRM1649b media was estimated to be 9 nM (Table S1). 3 PAHs, benzo[a]pyrene (BaP), benzo[e]pyrene (BeP) and benzo[k]fluoranthene (BkF) were identified from the certificate of analysis provided by NIST for testing in the Th17 polarization assay. These three PAHs are found at similar concentration in the SRM1649b, approximately 0.3 nM. The three PAHs were initially dissolved in DMSO as a primary stock solution and diluted further into a series of 3-fold secondary stock solutions in DMSO. These secondary stock solutions were then added to culture media at a final 1:1000 dilution, yielding a range of test concentrations between 0.1nM and 27 nM. DMSO diluted 1:1000 into culture media was used as a control. After 4 days of culture, analysis of culture supernatants by ELISA demonstrated that BkF stimulated much greater IL-17A expression than the other 2 PAHs as well as a mixture of the 3 PAHs (Figure 4C). Other preliminary studies have shown that higher doses of BaP (>50nM) do increase IL-17 expression in Th17 cultures. The effects of BkF were studied further by intracellular cytokine staining to determine the percent of IL-17A expressing cells in the culture. While the lower 0.3 nM dose of BkF increased the percent of IL-17^+^ CD4^+^ T cells, only the higher dose of 9 nM gave a statistically significant increase when BkF was added to Th17 polarizing cultures of B6 cells (Figure 4D). BkF had no effect on the percent of IL-17^+^ CD4^+^ T cells when using AHR^-/-^ cells.

### Th17 polarization enhancing activity is found in other PAH containing environmental samples

**Figure 5:**
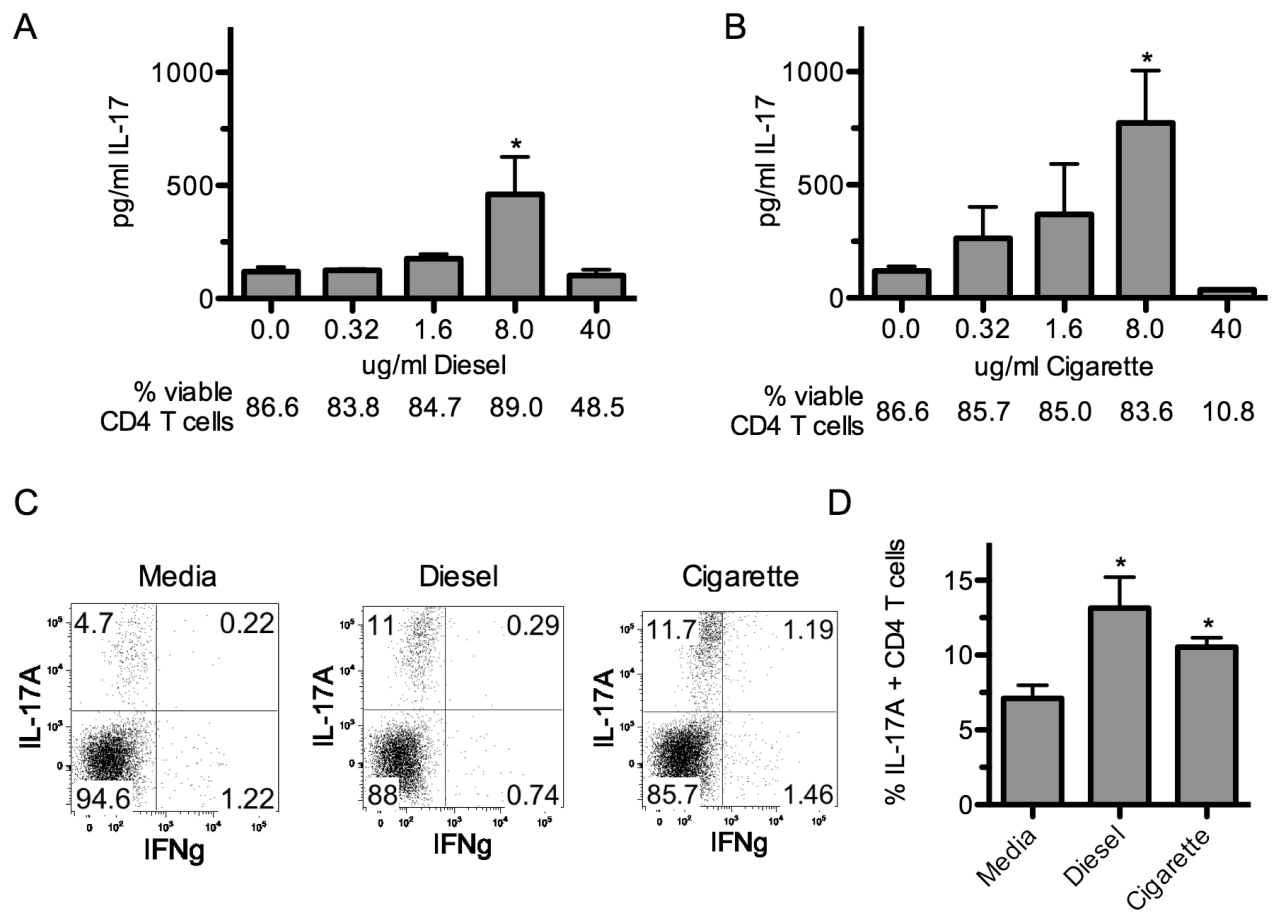
Th17 polarization enhancing activity is found in other PAH containing environmental samples. A - B: Naïve CD4^+^ T cells were isolated from the spleens of male B6 and cultured in Th17 conditions for 4 days in the presence of varying concentrations of diesel (A) and cigarette (B) extract. Culture media was harvested and the concentration of IL-17A was measured by ELISA. The percent of live/dead stain low CD4^+^ T cells is indicated below the appropriate extract concentration. C - D: Naïve B6 or AHR^-/-^ CD4^+^ T cells in Th17 conditions in the absence or presence of 8 μg/ml diesel or cigarette extract. Cells were harvested and subjected to intracellular cytokine analysis to determine the percent of IL-17A-expressing CD4^+^ T cells present after culture. For both ELISA and intracellular IL-17A results, data from 3 separate experiments was pooled and statistical comparison was made between the cultures with versus cultures without extract. *, p < 0.05.

 To determine whether Th17 polarization-enhancing activity found in SRM1649b could be demonstrated in other PM samples, extracts of 3 different freshly collected environmental samples, diesel exhaust, cigarette smoke, and wood smoke, were utilized in Th17 polarization assays. Dilutions of these extracts, titrating up to a dose of 40 µg/ml, were added into B6 Th17 polarizing cultures and the supernatants were assayed for IL-17A content. We chose this range because 40 μg/ml is the dose that corresponded to our strongest response with SRM1649b, and doses higher than this caused significant cell death. Wood smoke was extremely toxic to cells at several concentrations and showed no IL-17A upregulation at the lowest doses (data not shown). Both diesel (Figure 5A) and cigarette (Figure 5B) extracts enhanced IL-17 concentrations significantly at a concentration of 8 µg/ml. We additionally measured CYP1A1 and IL-22 expression at the 8 µg/ml dose and found that both of these were upregulated, consistent with the idea that these environmental samples contain AHR ligands (Figure S3). Extracts at the 40 µg/ml concentration resulted in greater cell death than seen in Th17 cultures with SRM1649b. Subsequent analysis in intracellular cytokine assays revealed that while 80 - 90 % of CD4^+^ T cells in Th17 cultures were viable on day 4 of culture (in controls or with SRM1649b), cultures containing diesel or cigarette extract at the 40 µg/ml concentration had only 50% and 10% viability respectively. Interestingly, while >80% of the viable cells in the diesel extract were AHR^+^ by flow cytometry, less than 20% of viable cells in the cigarette extract cultures were AHR^+^ (Figure S2). This might argue that in the setting of exposure to diesel, presence of the AHR protects cell viability in culture. Perhaps components of cigarette extract cause injury and death to cells independent of the AHR. Intracellular cytokine staining demonstrated that addition of 8 µg/ml diesel or cigarette extract to the culture gave rise to a significant increase in the percent of IL-17A^+^ cells (Figure 5C and D).

## Discussion

 A growing body of data strongly indicate that the cytokine IL-17 plays an important role in human airway disease. Studies in both animal models [31,32] and human subjects [33] have provided evidence that air pollutants can promote IL-17 expression in the lung. The data presented here support a hypothesis that PM can directly alter T cell function by enhancing Th17 polarization via an AHR-dependent mechanism. The Th17-promoting activity was contained in the organic fraction of SRM1649b and was independent of any cyp1a1-inhibitory activity that may also be contained within SRM1649b. The enhancement of Th17 differentiation was characterized by an upregulation in IL-17A, IL-22, and IL-23R. Increased IL-22 and IL-23R expression during Th17 differentiation has recently been suggested as a signature for pathogenic Th17 cells that can induce autoimmunity in animal models [34]. The implications of these previous findings and the current study are that air pollution can aggravate airway disease severity by promoting pathogenic Th17 cell differentiation through activation of the AHR. In addition to the effect in T cells, other mononuclear cell subsets can also express IL-17 and some of these cell types, including γδ-T cells [35], innate lymphoid cells [36] and mast cells [37] respond to AHR ligands by up regulating IL-17 expression. Thus, PM can potentially promote IL-17-mediated pathogenesis by multiple pathways. 

 PM includes a complex mixture of constituent chemicals. It is often classified by particle size, but this may oversimplify the molecular makeup of a sample or exposure [38–40]. While a number of components in PM may be responsible for aggravation of airway disease through a variety of mechanisms, we consider the role of PAHs in this manuscript as they are known AHR ligands. PAHs are ubiquitous contaminants in soil, are produced as byproducts of combustion of carbon-containing fuels including wood, coal, and diesel, as well as the burning of tobacco [41,42]. They have received considerable attention as agents of airway disease [9,43,44]. In figure 4, we explored a potential role for PAHs in the mechanism of enhancement of Th17 differentiation. We showed that the organic fraction of SRM1649b retained AHR activity and the ability to enhance this differentiation. In addition, we further showed that at least one PAH found in SRM1649b also enhanced Th17 differentiation. We then extended our data to fresh samples of diesel exhaust and cigarette smoke extract, and again identified enhancement of Th17. Further experimentation will be needed to corroborate this finding with a role for these pollutants in airway disease. 

 The mechanism through which AHR activation contributes to Th17 differentiation is poorly understood and somewhat controversial. While the AHR is expressed at greater levels in murine and human Th17 cells compared to other T cell subsets [18], it is not required for murine Th17 differentiation. Indeed, we have found that the baseline level of IL-17 protein expression was actually higher in AHR^-/-^ mice compared to B6 or AHR heterozygous littermates (Figure 3C). However, the up-regulation of IL-17 in response to PM is dependent on the presence of the AHR. One possible explanation is that the AHR serves as a repressor to IL-17 transcription. In this model, “unliganded” AHR is associated with an unknown transcription factor controlling IL-17 expression. After ligand binding, the AHR dissociates from this transcription factor which is now free to activate the promoter for IL-17 expression. In the AHR^-/-^ mouse, there is no repression of IL-17 transcription by the AHR, leading to elevated levels of this cytokine at baseline. This is consistent with the finding that AHR^-/-^ mice do not have obvious immunologic abnormalities at baseline, but when immunologically stressed (exposed to LPS [45,46] or certain gut bacteria [47]) they do fare worse than wild-type mice.

 We have developed the following theory regarding the role of the AHR as a sensor to environmental signals: When an individual is exposed to PM, mucosal defense mechanisms and mucociliary clearance of bacteria is altered, leaving the host at increased risk for pathologic infection. When high levels of these exposures are present, the AHR is activated, with generation of Th17 cells to fight bacterial insult, and secretion of IL-22, a unique cytokine secreted by immune cells that acts on local epithelial cells and engenders epithelial cell homeostasis, proliferation, and anti-microbial peptide production [48]. At the same time, activation of the AHR causes an upregulation of cytochrome P450 enzymes that metabolize the harmful toxicants. Initially this would be protective in nature, but over time sustained toxic exposures can lead to inflammation, fibrosis, and end-organ damage due to prolonged shifting of T cell differentiation towards an effector response. It is this concept that allows us to consider the role of the AHR in both protective and pathologic responses that will help in both understanding mechanisms of environmentally induced disease, and also allow targeting of this receptor for treatment.

 It does make sense that the acquired immune system is involved as an early responder in any environmental insult, particularly at interfaces with the outside world. Resident cells of these environments must have some way of responding to exposures that will put the organism at risk of invasion of endogenous bacteria and other pathology. The recent publications that identify the AHR as a critical component for the presence of innate cells of the gut and skin (γδ-T cells, intraepithelial lymphocytes, intestinal lymphoid follicles) highlight the role that the AHR might play in these interface organs [35,36,49,50]. These data support the importance of the AHR in the presence and response of sentinel cells of the immune system that respond to ligands generated by bacteria, food, and environmental toxins. This can be both protective and pathologic, depending on the inflammatory state and length of exposure, a finding that we think applies to the lung-environment interface as well. Parsing out the mechanistic differences that define how AHR activation alters IL-17 versus IL-22 expression in T cells as well as other cell types has the potential to define targets for new clinical therapies for airway disease.

## Supporting Information

Figure S1 **Elevated AHR expression in Th17 cultures is not limited IL-17A expressing cells.** Naïve CD4^+^ T cells were isolated from the spleens of male B6 mice and cultured in Th17 conditions for 4 days. Cells were stained with anti-AHR antibody as well as anti-IL-17A and IFN-γ antibodies during intracellular cytokine analysis. AHR expression for IL-17A^+^/ IFN-γ^-^ (thick line), IL-17A^-^/ IFN-γ^+^ (thin line) and IL-17A^-^/ IFN-γ^-^ (shaded) is shown.
(TIFF)

Figure S2 **Exposure to high levels of toxic pollutants leads to selective viability of AHR expressing T cells *invitro*.** Naïve CD4^+^ T cells were isolated from the spleens of male B6 mice and cultured in Th17 conditions for 4 days in the presence of 8 or 40 μg/ml diesel exhaust and cigarette smoke extract. Cells were harvested and subjected to intracellular cytokine analysis to determine the percent of live AHR and IL-17A-expressing CD4^+^ T cells present after culture.
(TIFF)

Figure S3 **Exposure to Diesel and Cigarette extract upregulates genes associated with AHR activation.** Naïve CD4+ T cells were isolated from the spleens of male B6 mice and cultured in Th17 conditions for 4 days in the presence of 8 μg/ml diesel exhaust and cigarette smoke extract. Total RNA was harvested and, the relative levels of CYP1A1 and IL-22 mRNA were determined by qRT-PCR. Data is relative to cell treated with anti-CD3/CD28 antibody stimulation but without added mIL-6 and huTGF-β. Fold increase results from cultures with extract were compared for significance with cultures without extract using the paired student’s t-test. *, p < 0.05.
(TIFF)

Table S1 (DOCX)
