# Supplementary figures and images for: Exposure to Atmospheric Particulate Matter Enhances Th17 Polarization through the Aryl Hydrocarbon Receptor
Source: PLoS One. 2013 Dec 11;8(12):e82545. doi: 10.1371/journal.pone.0082545 (PMC3859609; doi:10.1371/journal.pone.0082545)

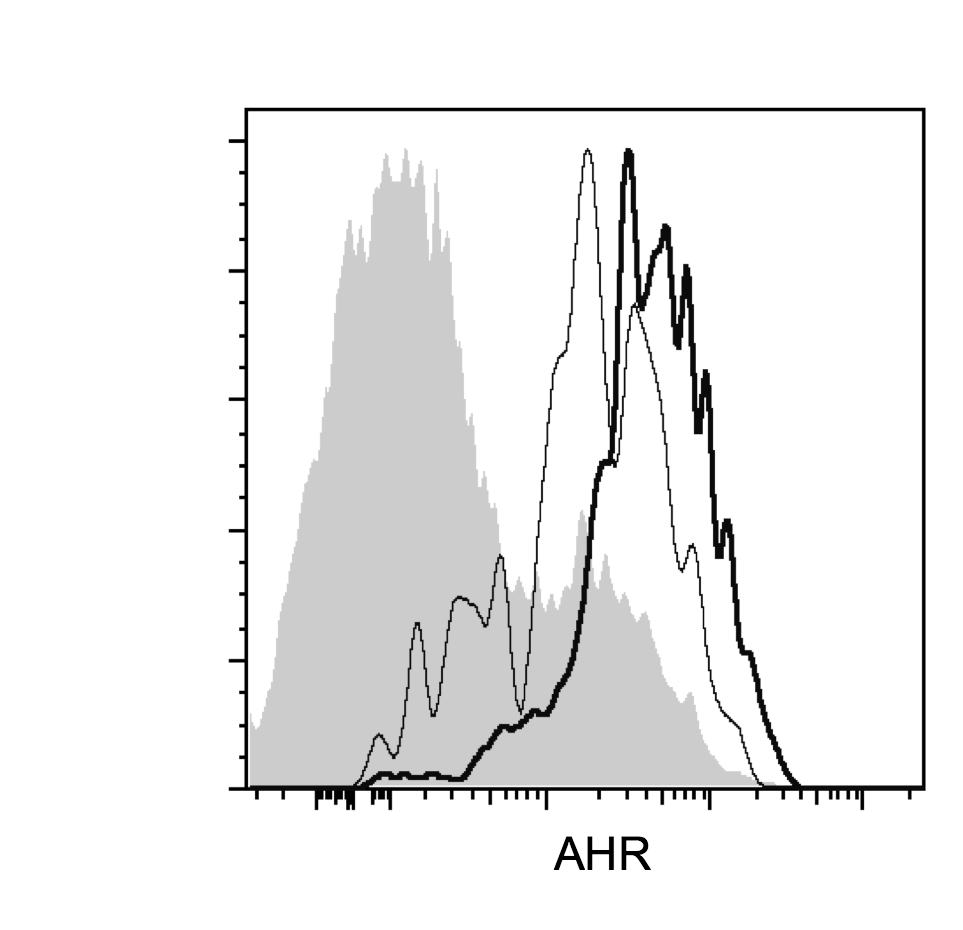

Supplement: Figure S1 — Elevated AHR expression in Th17 cultures is not limited IL-17A expressing cells. Naïve CD4+ T cells were isolated from the spleens of male B6 mice and cultured in Th17 conditions for 4 days. Cells were stained with anti-AHR antibody as well as anti-IL-17A and IFN-γ antibodies during intracellular cytokine analysis. AHR expression for IL-17A+/ IFN-γ- (thick line), IL-17A-/ IFN-γ+ (thin line) and IL-17A-/ IFN-γ- (shaded) is shown. (TIFF) [file pone.0082545.s001.tiff]

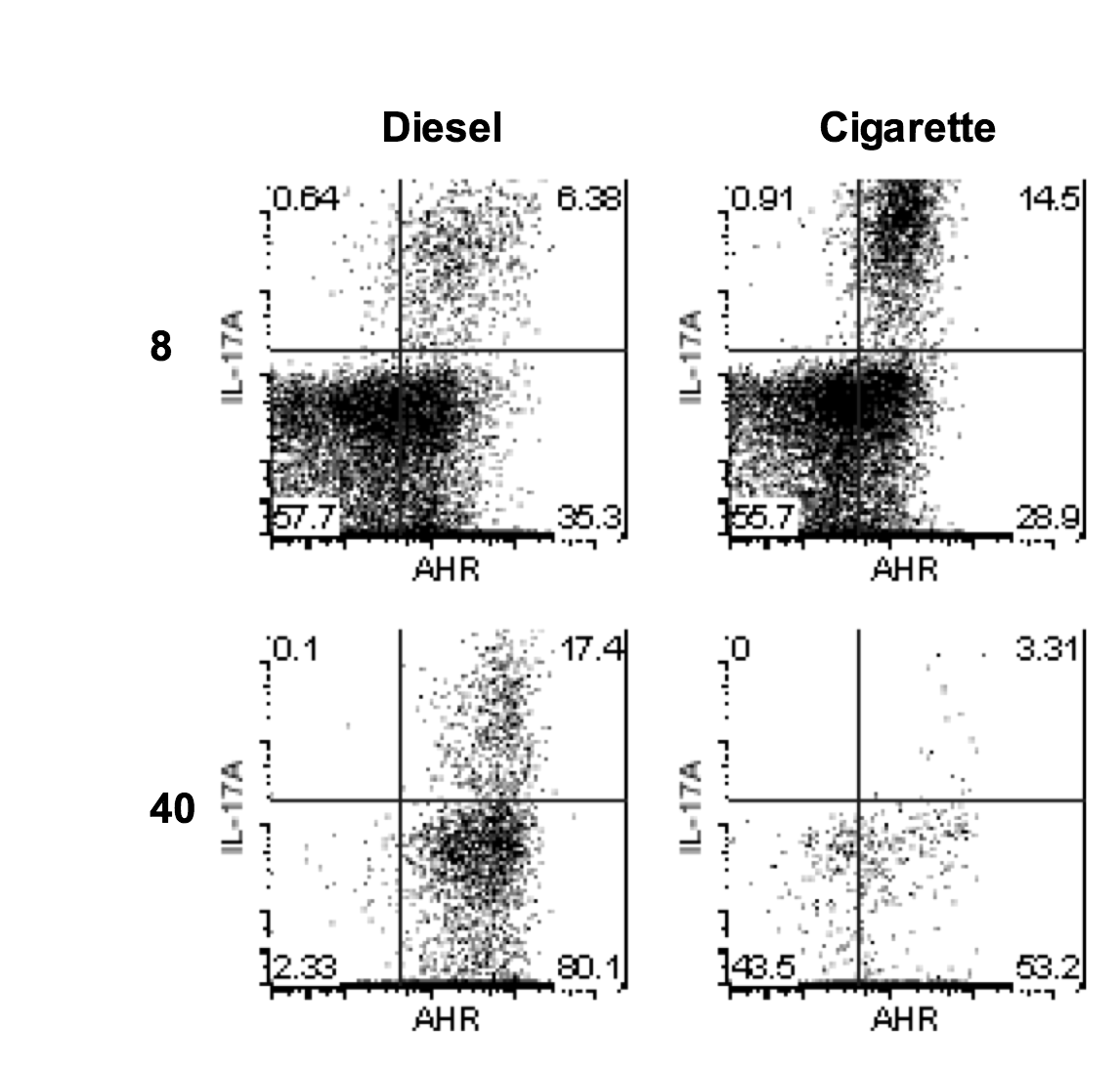

Supplement: Figure S2 — Exposure to high levels of toxic pollutants leads to selective viability of AHR expressing T cells invitro. Naïve CD4+ T cells were isolated from the spleens of male B6 mice and cultured in Th17 conditions for 4 days in the presence of 8 or 40 μg/ml diesel exhaust and cigarette smoke extract. Cells were harvested and subjected to intracellular cytokine analysis to determine the percent of live AHR and IL-17A-expressing CD4+ T cells present after culture. (TIFF) [file pone.0082545.s002.tiff]

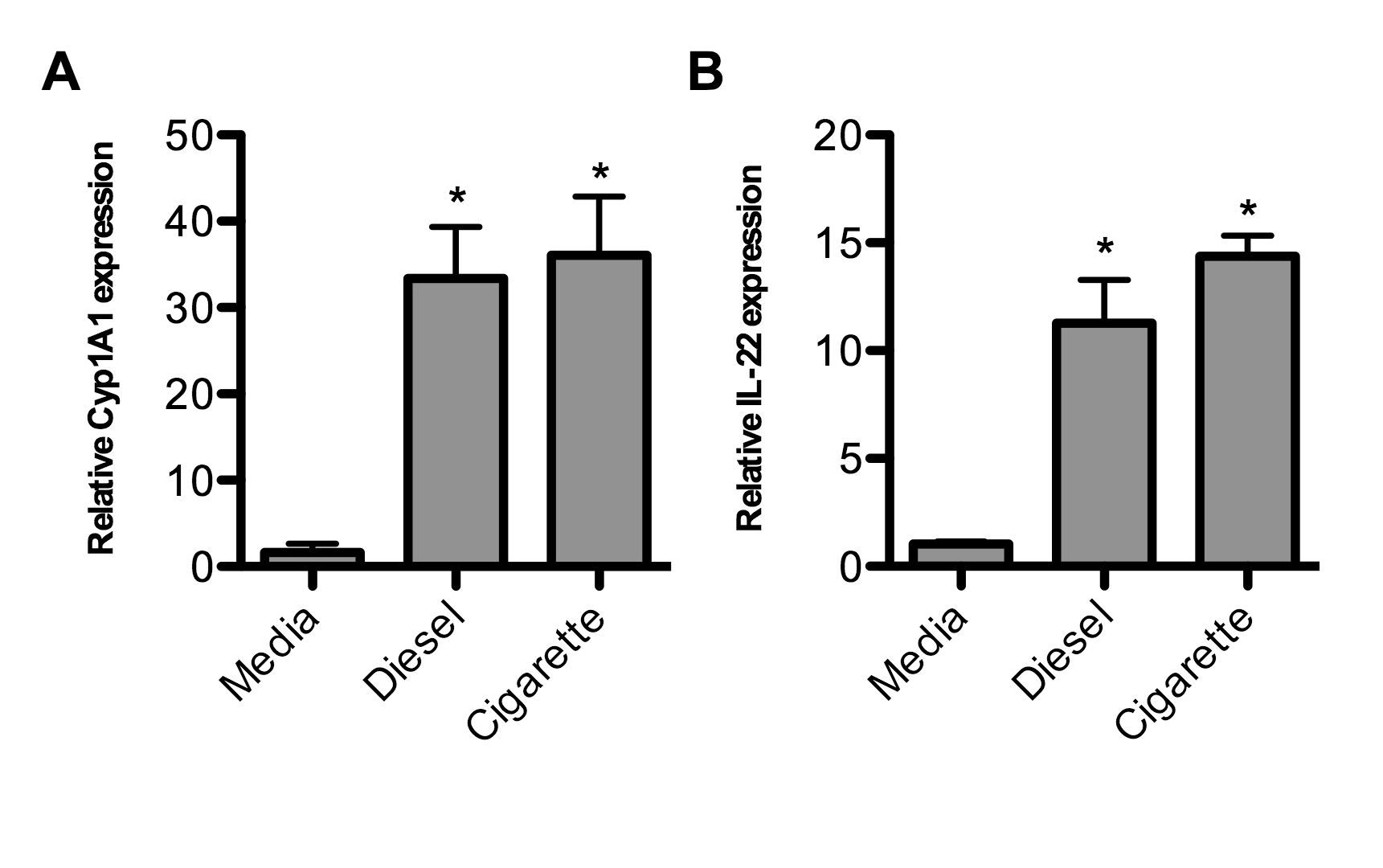

Supplement: Figure S3 — Exposure to Diesel and Cigarette extract upregulates genes associated with AHR activation. Naïve CD4+ T cells were isolated from the spleens of male B6 mice and cultured in Th17 conditions for 4 days in the presence of 8 μg/ml diesel exhaust and cigarette smoke extract. Total RNA was harvested and, the relative levels of CYP1A1 and IL-22 mRNA were determined by qRT-PCR. Data is relative to cell treated with anti-CD3/CD28 antibody stimulation but without added mIL-6 and huTGF-β. Fold increase results from cultures with extract were compared for significance with cultures without extract using the paired student’s t-test. *, p < 0.05. (TIFF) [file pone.0082545.s003.tiff]
