# Supplementary material for: Exposure to Atmospheric Particulate Matter Enhances Th17 Polarization through the Aryl Hydrocarbon Receptor
Source: PLoS One. 2013 Dec 11;8(12):e82545. doi: 10.1371/journal.pone.0082545 (PMC3859609; doi:10.1371/journal.pone.0082545)
Supplement: Table S1 — (DOCX) [file pone.0082545.s004.docx]

Table S1. Estimated nM concentrations of selected PAHs contained in 40 ug/ml SRM1649b

| Compound | MW | pg/μg SRM1649b | nM |
| --- | --- | --- | --- |
| Acephenanthrylene | 2.02.25 | 0.18 | 0.04 |
| Retene | 234.34 | 0.25 | 0.04 |
| Perylene | 252.31 | 0.61 | 0.10 |
| Benzo(j)fluoranthene | 252.31 | 1.73 | 0.27 |
| Anthracene | 178.23 | 0.40 | 0.09 |
| Picene | 278.35 | 0.39 | 0.06 |
| Dibenzo(ae)pyrene | 302.37 | 0.54 | 0.07 |
| Benzo(GHI)fluoranthene | 226.27 | 0.88 | 0.16 |
| Dibenz(ah)anthracene | 278.35 | 0.29 | 0.04 |
| Benz(a)anthracene | 228.29 | 2.09 | 0.37 |
| Benzo(a)pyrene | 252.31 | 2.47 | 0.39 |
| Coronene | 234.34 | 2.83 | 0.38 |
| Indeno(1,2,3-cd)pyrene | 276.33 | 2.96 | 0.43 |
| Benzo(k)fluoranthene | 252.31 | 1.75 | 0.28 |
| Chrysene | 228.29 | 3.01 | 0.53 |
| Phenanthrene | 178.23 | 3.94 | 0.88 |
| Benzo(e)pyrene | 252.31 | 2.97 | 0.47 |
| Benzo(GHI)perylene | 226.27 | 3.94 | 0.57 |
| Pyrene | 202.25 | 4.78 | 0.95 |
| Benzo(b)fluoranthene | 252.31 | 5.99 | 0.95 |
| Fluoranthene | 202.25 | 6.14 | 1.21 |
